# Supplementary material for: The First Two Years of COVID-19 Hospitalization Characteristics and Costs: Results from the National Discharge Registry
Source: Healthcare (Basel). 2024 May 7;12(10):958. doi: 10.3390/healthcare12100958 (PMC11121639; doi:10.3390/healthcare12100958)
Supplement: Supplementary file 1 [file healthcare-12-00958-s001.zip › healthcare-2923286-supplementary.pdf]

Table S1: Median age of COVID-19 hospitalized patients and population by macro-area and year. Italy 2020-2021

Year	macro-area	Median Age (years)		
		Hospitalizations outcome		Population
		alive	deaths	
2020				
	Nord-west	67.7	78.0	48.0
	Nord-east	70.4	78.6	47.8
	Centre	66.2	78.0	47.9
	South	61.5	76.7	45.5
	Major Islands	61.3	77.0	46.4
	Overall	67.3	78.0	47.2
2021				
	Nord-west	67.6	78.1	48.2
	Nord-east	68.5	78.4	48.0
	Centre	62.2	77.7	48.2
	South	60.9	76.1	46.0
	Major Islands	61.0	76.8	46.9
	Overall	65.8	77.7	47.5

Table S2 Population, COVID-19 hospitalization rates and percentage of hospital deaths and males by region and year. Italy 2020-2021

macro-area	Region	2020				2021			
		Patients[1]	hosp. rate[2]	deaths (%)	Male (%)	Patients[1]	hosp. rate[2]	deaths (%)	Male (%)
Northwest									
	Piedmont	32,073	8.6	27.6	57.2	28,862	6.8	20.4	55.4
	Aosta Valley	1,063	9.8	24.6	53.0	1,071	8.6	10.0	50.8
	Lombardy	82,259	9.5	23.8	58.9	59,871	6.0	17.1	55.7
	Liguria	12,185	9.3	25.5	55.0	12,465	8.2	15.4	54.3
Northeast									
	Trentino-Alto Adige	6,660	7.2	18.0	56.5	6,906	6.4	13.5	55.6
	Veneto	22,505	5.3	22.4	56.6	24,729	5.1	18.9	56.6
	Friuli-Venezia Giulia	5,608	5.4	22.4	55.1	9,235	7.7	22.2	56.4
	Emilia-Romagna	34,733	9.0	22.1	53.9	35,188	7.9	17.9	54.0
Centre									
	Tuscany	14,823	4.6	19.3	54.9	21,403	5.8	16.2	55.3
	Umbria	2,915	3.9	20.5	56.3	4,986	5.8	17.0	54.7
	Marche	6,163	4.7	26.9	55.8	8,100	5.4	20.1	55.7
	Lazio	27,827	5.6	18.4	55.4	42,116	7.3	14.1	53.6
South									
	Abruzzo	5,326	4.8	22.8	58.1	7,343	5.7	17.4	56.3
	Molise	717	2.8	25.8	59.0	1,133	3.8	28.9	58.3
	Campania	12,908	2.6	24.1	61.1	19,628	3.5	24.6	55.1
	Apulia	12,836	3.8	20.0	57.9	18,601	4.7	21.0	57.2
	Basilicata	1,097	2.3	23.7	55.9	1,992	3.7	19.5	55.3
	Calabria	2,256	1.4	16.8	58.1	5,082	2.7	19.4	56.8
Major Islands									
	Sicily	11,645	2.8	18.1	54.0	20,432	4.2	19.3	53.7
	Sardinia	3,301	2.4	19.7	55.7	4,305	2.7	18.9	54.8

[1] there are 3275 patients with foreign residence
[2] hospitalization rate

Table S3: Percentages of acute care, surgical treatments, and day hospital of COVID-19 hospitalization in Italy (2020-2021)

anno	mal	Acute	surgery	Day H.
2020	COVID-19	93.9	6.7	1.1
2021	COVID-19	93.7	8.4	2.9
